# Diagnosing of primary cutaneous amyloidosis using dermoscopy and reflectance confocal microscopy

**DOI:** 10.1111/srt.13143

**Published:** 2022-02-21

**Authors:** Wang Lei, Xu Ai‐E

**Affiliations:** ^1^ Department of Dermatology Third People's Hospital of Hangzhou Hangzhou China

**Keywords:** amyloidosis, confocal, dermoscopy, microscopy

## Abstract

**Background:**

Primary cutaneous amyloidosis (PCA) is apruritic and potentially disfiguring disorder; this disease is usually diagnosed clinically due to its common occurrence. However, for cases with atypical presentations or for those physicians not familiar with PCA, the diagnosis can be a challenge.

**Objective:**

To observe the characteristics of PCA under dermoscopy and reflectance confocal microscopy (RCM) in order to gain experience and reference for clinicians to facilitate diagnosis.

**Methods:**

The typical lesions of 110 patients with primary cutaneous amyloidosis were observed by dermoscopy and RCM, and scanning results were recorded. Thirty patients followed by complete excision for histopathological analysis.

**Results:**

A total of 110 patients with clinically diagnosed PCA were enrolled. Forty‐seven patients had lesions consistent with macular amyloidosis and 63 with lichen amyloidosus. The dermoscopic findings of PCA shared a common feature, each ‘macule’ was composed of a central hub pattern surrounded by brownish pigmentation, The pattern of the central hub could be brown, white, scar‐like and structureless area. RCM features of total patients consisted of dermal papilla present cloud‐like agglomerate which are high refractive index.

**Conclusions:**

Dermoscopy and reflectance confocal microscopy can be used in the diagnosis of PCA, which can provide a basis for doctors to diagnose.

## 1 INTRODUCTION

Primary cutaneous amyloidosis (PCA) is a pruritic and potentially disfiguring disorder; this disease may impact greatly a patient's social and emotional status. The most common subtypes are macular amyloidosis (MA) and lichen amyloidosus (LA).^1^, ^2^ PCA is usually diagnosed clinically due to its common occurrence. However, for cases with atypical presentations or for those physicians not familiar with PCA, the diagnosis can be a challenge.

Non‐invasive techniques, including reflectance confocal microscopy (RCM) and dermoscopy, and other techniques are already in use for assisting diagnosis and monitoring disease development. Dermoscopy is an important non‐invasive technique to diagnose pigmented and non‐pigmented cutaneous lesions, which can visualize the pigmented structures of the epidermis, the dermo‐epidermal junction, and the dermis.^3^, ^4^, ^5^ RCM is a widely used tool that enables real‐time, visualization of skin layers on a horizontal plane, and serial optical sections of the lesions from the epidermis to the depth of 200–350 μm. Its high cellular resolution is close to that of conventional histology.^6^, ^7^ This feature is particularly useful in head and neck examinations, where it potentially avoids surgical operations in anatomically dangerous areas and areas that affect aesthetics.

The dermoscopy and RCM features of PCA have not been adequately described in the literature.^8^ We used dermoscopy combined with RCM to diagnose 110 patients of PCA. These imaging features can be used as a reference for clinicians to diagnose PCA. We describe dermoscopy features of PCA in these 110 cases. We also describe RCM appearance of PCA.

## 2 MATERIALS AND METHODS

### 2.1 Patients

One hundred ten patients with a previously established diagnosis of MA or LA were recruited from the Department of Dermatology, The Third People's Hospital of Hang Zhou, China, from June 2015 and October 2020 after giving informed consent. None of the patients was under treatment in the month prior to the study. Thirty patients were imaged using dermoscope and in vivo RCM, followed by complete excision for histopathological analysis. Lesions without a definite histopathological diagnosis of PCA were excluded from the study.

### 2.2 Dermoscopic imaging

Baseline clinical images were taken with a Sony DSC‐W630 digital camera (Sony Corp., Tokyo, Japan), and digital dermatoscopic images of the lesions were obtained by using a Heine Delta 20 dermatoscope (Heine, Herrsching, Germany) (10‐fold magnification) mounted on a Canon EOS 600D camera (Canon Corp., Tokyo, Japan).

### 2.3 RCM imaging

A commercially available in vivo RCM device (Vivascope 1500; Lucid Inc., Rochester, NY, USA) was used for imaging. The instrument operates with a diode laser at an 830 nm wavelength and a laser power < 35 mW at the tissue level. This system provides high‐resolution images (approximately 1−2 lm in the lateral dimension and 4−5 lm in the axial ones) to a depth of 200−350 μm in vivo (from the epidermis down to the superficial reticular dermis).

## 3 RESULT

### 3.1 Clinical and histologic features

A total of 110 clinically diagnosed PCA patients were enrolled. Forty‐seven patients had lesions consistent with MA, and 63 with LA. Thirty patients, including 11 cases of MA and 19 cases of LA, underwent skin biopsy and their histopathological findings were compatible with PCA, including basal hyperpigmentation, pigment incontinence, and amyloid deposition in the papillary dermis. In addition, compact orthohyperkeratosis and acanthosis were also found for most LA specimens.

### 3.2 Dermoscopic features

**FIGURE 1:**
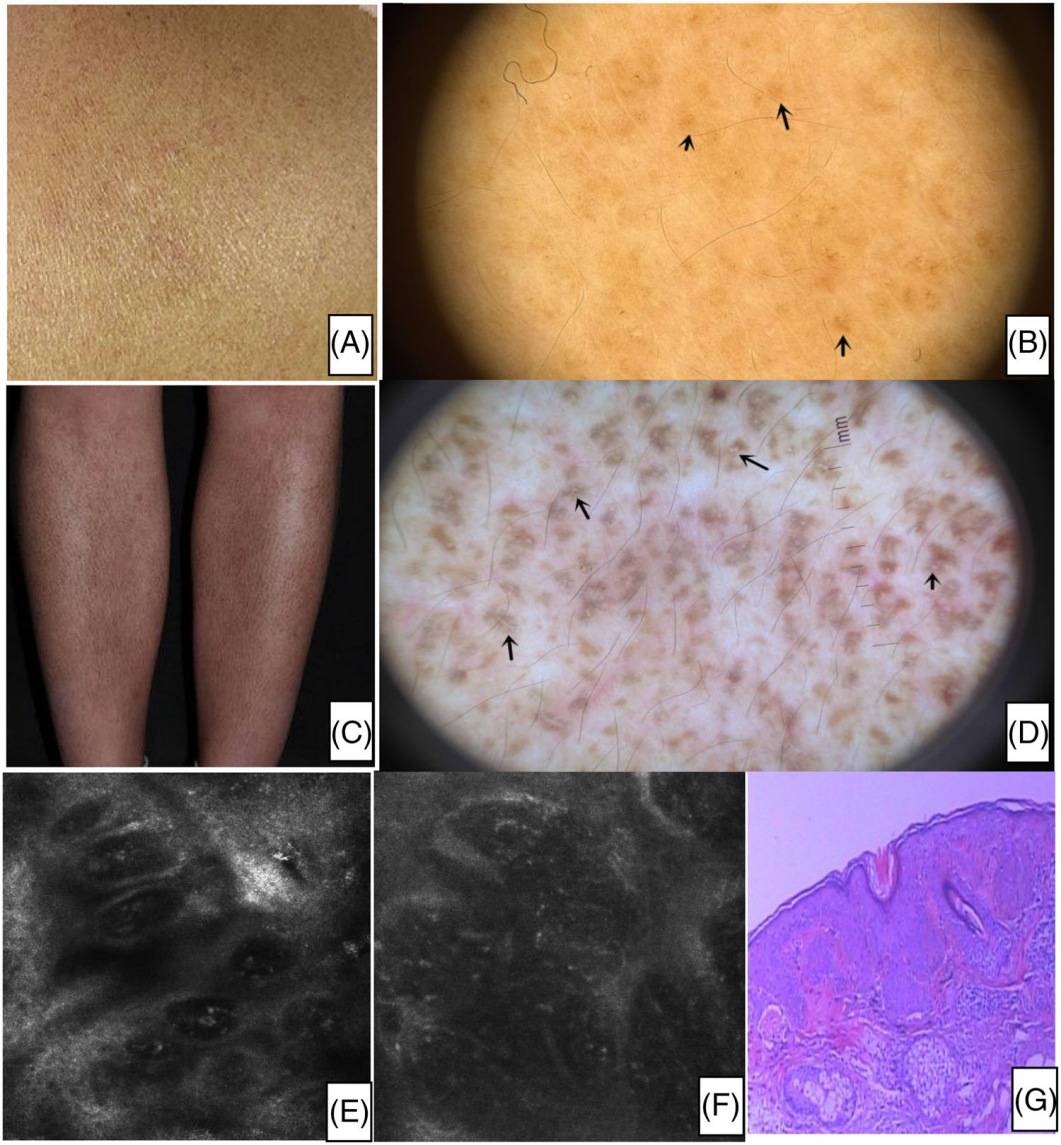
(A) Clinical picture: multiple rippling brownish macules on the scapular area of the back. (B) Dermoscopic feature: The dermoscopic findings revealed multiple uniform small central hubs with brown fine streaks radiating from the centre. The colour of the central hubs was brown in these cases. (C) Clinical picture: multiple rippling brownish macules on the lower right legs. (D) Dermoscopic feature: The dermoscopic pattern showed each white central hub surrounded by venation‐like extension of pigmentation. (E) Rectance confocal microscopy basic image shows papillary rims, which usually are visible in normal skin, are obscured, total or partial obliteration of the ring‐like structures around the dermalpapillae. (F) Rectance confocal microscopy basic image shows melanophages, visible as brightly refractile, plump, oval to stellate‐shaped cells, infiltrate in the layer of superficial dermis. (C) Histopathological image shows liquefaction of basal cells and incontinence of pigment

**FIGURE 2:**
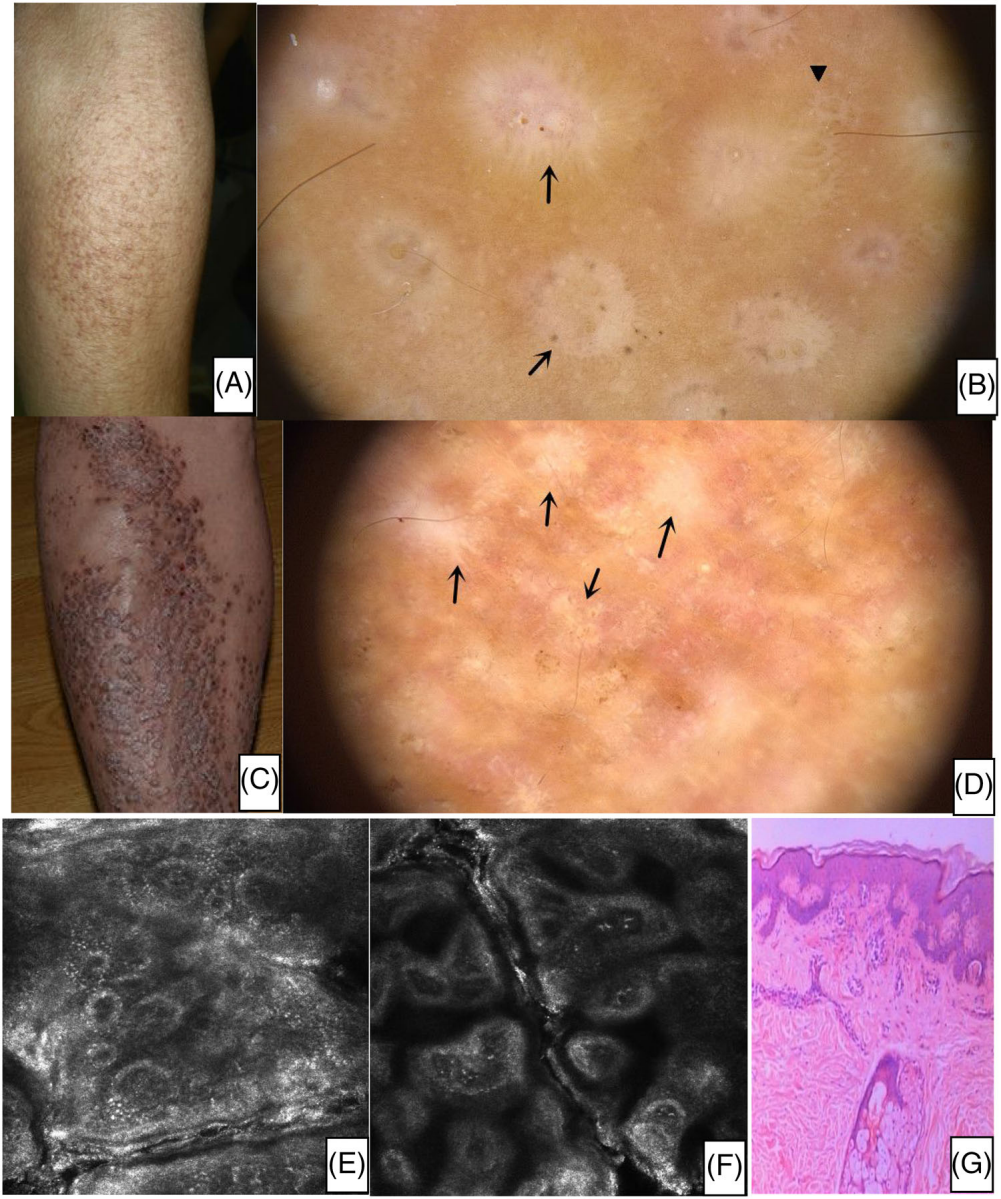
(A) Clinical picture: The clinical picture of a patient with lichen amyloidosus. (B) Under dermoscopy, each papule showed a scar‐like centre surrounded by some brownish pigmentation (arrows) or a rim of white collaret (arrowhead). The entire picture resembled a volcanic crater. (C) Clinical picture: hyper‐pigmented lichenified papules with a predilection for the left leg. (D) Dermoscopic feature: In this patient with larger papules, the central hub was completely replaced by a scar‐like structureless area. (E) Rectance confocal microscopy basic image shows the increased melanin in the basal cell layer of the primary cutaneous amyloidosis (PCA) lesion. (F) Rectance confocal microscopy basic image shows dermal papilla that presents cloud‐like agglomerates, which are of high refractive index. There were some dotted substance, coliform substance, rope, and so on in the agglomerate. The annular structure of papillary dermis increases and appears pleomorphism. (C) Histopathological image shows hyperkeratosis, parakeratosis, acanthosis, and variable melanin pigmentation in basal layer, and enlarged dermal papilla deposition of amyloid

The dermoscopic findings of PCA shared a common feature, each 'macule' was composed of a central hub pattern surrounded by brownish pigmentation. The pattern of the central hub could be brown, white, scar‐like, and structureless area. Eleven patients showed brown central hubs ware all MA (Figure 1A,B), white central hubs ware 43 patients (Figure 1C,D), and there were seven cases of LA and 36 cases of MA. In 33 cases, the central hub was a scar‐like centre (Figure 2A,B), and the other 23 (Figure 2C,D) cases showed completely structureless area. A variety of morphologic pigment structures (dotted, globular, streaks, radial, and leaf‐like extensions, etc.) were observed in all lesions around the central area. Dermoscopic features are summarized in Table 1.

**TABLE 1:** Patient numbers of primary cutaneous amyloidosis with different clinical types and dermoscopic central hub patterns

**Clinical type**	**Central hub pattern**	***n***
Lichen amyloidosus (*n* = 63)	White	7
	Brown	0
	Scar‐like	33
	Structureless area	23
Macular amyloidosis (*n* = 47)	White	36
	Brown	11
	Scar‐like	0
	Structureless area	0

### 3.3 RCM features

The most characteristic RCM features of total patients consisted of dermal papilla present cloud‐like agglomerate which are high refractive index (*n* = 110). There were some dotted substances, coliform substance, rope, and so on in the agglomerate. The increased melanin in the basal cell layer of the PCA lesion. Dermal papilla present cloud‐like agglomerate which are high refractive index. There were some dotted substance, coliform substance, rope and so on in the agglomerate. The annular structure of papillary dermis increase and appear pleomorphism (Figure 2E,F,G). At the level of the dermal‐epidermal junction, papillary rims, which usually are visible in normal skin, are obscured, total or partial obliteration of the ring‐like structures around the dermal papillae (*n* = 73). Melanophages are visible as brightly refractile, plump, oval‐to stellate‐shaped cells, infiltrate in the layer of dermis, associated with a few scattered round‐to‐polygonal, mildly refractive cells (Figure 1E,F,G). When RCM longitudinal detection scan,from the epidermis down to the superficial reticular dermis showed hyperkeratosis (*n* = 81), acanthosis (*n* = 60), and variable melanin pigmentation in the basal layer (*n* = 73).

Reflectance confocal microscopy and histopathological findings are presented in Table 2.

**TABLE 2:** Reflectance confocal microscopy (RCM) and histopathological features of 30 cases of primary cutaneous amyloidosis (PCA) (case)

	Lichen amyloidosus (*n* = 19)		Macular amyloidosis (*n* = 11)		Number of cases (*n* = 30)	
Observed indicator	RCM	Histopathology	RCM	Histopathology	RCM	Histopathology
Hyperkeratosis	15	15	7	7	22	22
Acanthosis	11	11	4	4	15	15
Variable melanin pigmentation in basal layer	13	13	7	7	20	20
Amyloid deposition in the papillary dermis	19	19	11	11	30	30
Enlarged dermal papilla	10	10	2	2	12	12
Incontinence of pigment	10	13	10	10	20	23
Lymphocytes infiltration	6	6	5	5	11	11

## 4 DISCUSSION

In this study, we report the dermoscopy and RCM characteristics of PCA. The most clinical lesions of PCA showed brownish colour and dermoscopy has been demonstrated to be an ideal tool to show the magnified characteristics of pigmented skin disorders. The typical dermatoscopic expression patterns of PCA are different central hubs. In this study, all lesions have central hubs pattern: brown, white, scar‐like, and structureless area. There were only two central patterns of brown and white in MA lesions, and the brown central hubs corresponded to the normal histopathological cuticle. Mild hyperkeratosis and acanthosis are seen as a white central area under dermoscopy. Dermoscopic images show the pattern of central hubs ware scar‐like and structureless area exists in LA, because of compact orthohyperkeratosis and acanthosis. All central hubs pattern is surrounded by various configurations of pigmentation, including dotted, globular, streaks, radial, and leaf‐like extensions. In addition, we used the RCM explanation for the different arrangements of pigmentation, found pigmentation ware dotted, globular, showed pigmented incontinence under RCM, linear, radial and leaf‐like extensions ware the basal hyperpigmentation increased, and pigment distribution more focused on the same level in the organization.

Reflectance confocal microscopy has been used for the evaluation of varied skin conditions, showing a good correlation with conventional histopathologic examination.^9^, ^10^, ^11^ In our study, RCM demonstrated to be able to show the details of specimens, in high accordance with the results of optical microscopy. We have examined PCA patients by RCM and correlated them with corresponding histopathological findings, aiming to identify reliable features for a preliminary evaluation of the effectiveness of RCM for the diagnosis of PCA. Table 2 summarizes these features. Hyperkeratosis and irregular acanthosis were the common epidermal findings of PCA. The most characteristic features were the presence of amyloid deposition, which was defined as dermal papilla present, cloud‐like agglomerate which are high in refractive index under RCM. There were some dotted substance, coliform substance, rope, and so on in the agglomerate. The annular structure of the papillary dermis increases and appears pleomorphic. Compared with histopathology, pigmented incontinence was not found in three cases of LA under RCM, which may be due to the limited depth of RCM detection. Our research showed that the degree of hyperkeratosis, spinous layer hypertrophy, and amyloid deposition in LA was more obvious than that in MA, this is consistent with previous studies.^12^ Therefore, in most cases LA dermoscopic images show the pattern of central hubs ware whitish scar‐like centres and structureless area.

**FIGURE 3:**
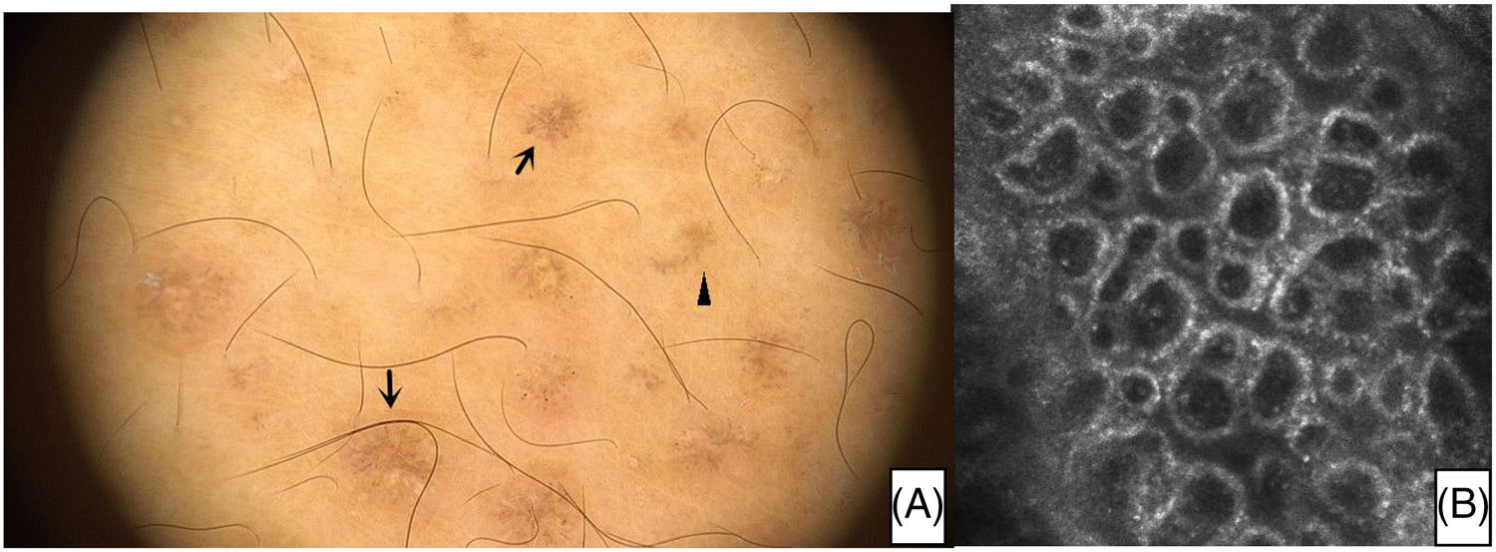
(A) Dermoscopic examination showed characteristic central hubs with radial streaks (arrows) and leaf‐like extensions (arrowhead). (B) Rectance confocal microscopy basic image shows dermal papilla that presents cloud‐like agglomerate, which are of high refractive index, basal hyperpigmentation increased, and pigment distribution is more focused on the same level in the organization

The diagnosis of PCA is usually straightforward, especially if the morphology and distribution of lesions are typical. However, if the area of involvement is located over an unusual site, it becomes difficult to make an accurate diagnosis. However, if the area of involvement is located over an unusual site, it becomes difficult to make an accurate diagnosis. For instance, one of our patients presented with multiple pinhead‐sized pigmented dots on his face. As the scalp is seldom affected by PCA, skin biopsy was performed and MA was then proved. His dermoscopic examination showed characteristic central hubs with radial streaks and leaf‐like extensions pigmentation. These dermoscopic pictures are strikingly similar to the ‘spoke wheel’ and ‘maple leaf‐like areas’ patterns, which are two of the six dermoscopic criteria to diagnose basal cell carcinoma.^13^ However, RCM basic image shows dermal papilla that presents cloud‐like agglomerate, which are of high refractive index, basal hyperpigmentation increased, and pigment distribution is more focused on the same level in the organization (Figure 3). Therefore, we suggest that RCM can be used to further assist diagnosis when there is doubt in using dermatoscopy.

Reflectance confocal microscopy may represent a real‐time, non‐invasive aid to the clinical diagnosis of PCA. However, it limited depth of detection, furthermore might be difficult to distinguish different subtypes of interface dermatitis, for example Riehl's melanosis versus lichen planus. This seems to be due to the subtle microscopical differences between these diseases on the superficial layers, as visualization of deeper structures is important for the differential diagnosis. Dermoscopic features add new diagnostic criteria for differentiation of PCA. Both reflectance modes offer different information that can be combined to improve diagnostic accuracy, but clinical evaluation should not be neglected.

## 5 CONCLUSION

Taken together, this study highlights the dermoscopic and confocal microscopic features of PCA. Dermoscopy showed brown, white, scar‐like, and structureless area central hubs surrounded by various configurations of pigmentation. We believe that dermoscopy and RCM are of great value in the non‐invasive examination of PCA and combined use of them can make the diagnosis of PCA more accurate.
